# The Role of Cognitive Behavioural Therapy, Family Therapy and Psychopharmacological Interventions in Internet Gaming Disorder: A Systematic Review

**DOI:** 10.1192/bjo.2024.248

**Published:** 2024-08-01

**Authors:** Sathyan Soundara Rajan, Gaurav Uppal, Khushboo Kansal, Ugo Okafor, Asha Dhandapani

**Affiliations:** 1 BCUHB, Wrexham, United Kingdom; 2 Satyam Hospital, Ludiana, India; 3 Nottinghamshire Healthcare NHS Trust, Nottingham, United Kingdom; 4 Mater Misericordiae Hospital, Dublin, Ireland

## Abstract

**Aims:**

Internet gaming disorder (IGD) is a recognised mental health condition characterised by impulsive gaming, where gaming takes precedence over all other activities and negatively impacts the life of a person. IGD has an estimated prevalence of 2–5% of all mental health disorders. Limited research exists on the treatment effects of various therapeutic interventions for gaming disorder, highlighting the need for comprehensive investigations of evidence-based approaches and to improve intervention strategies.

This systematic review aims to identify most of the intervention studies on internet gaming disorder using a control group, to determine the effect of the interventions and to examine moderators for these interventions.

**Methods:**

We reviewed available treatment interventions for children and adolescents. A search on Pubmed central, PsycINFO, Embase, MEDLINE, Cochrane, CINAHL and Google Scholar Library was conducted. Various interventions, whether individual or group-based, incorporate Cognitive Behavioural Therapy (CBT), family therapy and pharmacological treatments for gaming disorder and these were selected for this review among all the other interventions examined. Some exclusively use CBT, while others combine it with different treatments. This includes both online and in-person CBT, encompassing behavioural including limited exposure and cognitive elements.

The comprehensive search resulted in 113 studies from 2018–2023 and we ended up with 25 studies by excluding studies according to the exclusion criteria.

**Results:**

This systematic review identified a total of 113 studies, of which 25 studies were finally selected and were included. It examined interventions for internet gaming disorder (IGD). Cognitive Behavioural Therapy (CBT), Family Therapy, and Psychopharmacological treatments were assessed across diverse studies.

Findings indicate significant improvements post-intervention, with CBT and family therapy showing promising results in reducing IGD symptoms. Pharmacotherapy combined with psychotherapy emerged as the most effective treatment option. The study underscores the need for multifaceted approaches in addressing IGD, contributing valuable insights for future treatment strategies.

**Conclusion:**

The review highlights promising outcomes for Internet Gaming Disorder interventions, with Cognitive Behavioural Therapy and Family Therapy demonstrating effectiveness. Combining pharmacotherapy with psychotherapy is most beneficial, emphasising the importance of comprehensive treatments needed for IGDs.

